# Sex-Based Differences in Asthma among Preschool and School-Aged Children in Korea

**DOI:** 10.1371/journal.pone.0140057

**Published:** 2015-10-06

**Authors:** Yeonsoo Jang, Anna Shin

**Affiliations:** 1 College of Nursing, Mo-Im Kim Nursing Research Institute, Yonsei University, Seoul, Korea; 2 Department of Public Health, College of Medicine, Yonsei University, Seoul, Korea; Institute for Health & the Environment, UNITED STATES

## Abstract

The purpose of this study was to explore risk factors related to asthma prevalence among preschool and school-aged children using a representative national dataset from the Korea National Health and Nutrition Examination Survey (KNHANES) conducted from 2009–2011. We evaluated the demographic information, health status, household environment, socioeconomic status, and parents’ health status of 3,542 children aged 4–12 years. A sex-stratified multivariate logistic regression was used to obtain adjusted prevalence odds ratios (ORs) and 95% confidence intervals after accounting for primary sample units, stratification, and sample weights. The sex-specific asthma prevalence in the 4- to 12-year-old children was 7.39% in boys and 6.27% in girls. Boys and girls with comorbid atopic dermatitis were more likely to have asthma than those without atopic dermatitis (boys: OR = 2.20, p = 0.0071; girls: OR = 2.33, p = 0.0031). Boys and girls with ≥1 asthmatic parent were more likely to have asthma than those without asthmatic parents (boys: OR = 3.90, p = 0.0006; girls: OR = 3.65, p = 0.0138). As girls got older, the prevalence of asthma decreased (OR = 0.90, p = 0.0408). Girls residing in rural areas were 60% less likely to have asthma than those residing in urban areas (p = 0.0309). Boys with ≥5 family members were more likely to have asthma than those with ≤3 family members (OR = 2.45, p = 0.0323). The factors related to asthma prevalence may differ depending on sex in preschool and school-aged children. By understanding the characteristics of sex-based differences in asthma, individualized asthma management plans may be established clinically.

## Introduction

Asthma is a common chronic respiratory disease with serious clinical outcomes. The prevalence of this noncommunicable disease has increased, and asthma currently affects approximately 235 million people and 14% of children worldwide [1, 2]. According to studies of the noncommunicable disease burden in South Korea conducted in 2002 and 2007, asthma was ranked as the fourth and fifth leading cause of disease burden, and among children and adolescents, asthma caused the greatest disease burden [3–5].

Aggravation of asthmatic symptoms such as breathlessness and coughing can interrupt a patient’s daily routine and work competency. Furthermore, frequent symptom recurrences can result in socioeconomic burdens on patients and a reduced quality of life. Particularly, it is important to provide timely treatment for childhood asthma because this disease can persist throughout adulthood if not properly treated [3]. The Korean Youth Risk Behavior Web-based Survey showed that 24% of all school days missed by children and adolescents were related to asthma, indicating that asthma has a major impact on their quality of life and learning ability [6].

Previous studies have suggested sociodemographic characteristics, family history, obesity, smoking, and other medical conditions as factors related to asthma prevalence [7–10]. Some studies have investigated sex-related differences in the associations between these factors and asthma prevalence [11, 12]. Asthma is more common and more severe in prepubertal boys and boys <18 years of age compared with girls of the same age, but the asthma prevalence and severity increase significantly in women after puberty [13, 14]. The mechanism of and related factors associated with asthma development largely remain uncertain; an understanding of sex-based differences is needed to devise effective and individualized asthma management strategies. However, few studies have used nationwide data to investigate sex-based differences in asthma prevalence among preschool and school-aged children.

Many cases of chronic asthma begin in preschool age. In some cases, the asthmatic symptoms disappear in early school age but in other cases may recur or become exacerbated during school age. Symptoms in the first 3 years of life are often transient, and 3 of 4 asthmatic patients outgrow asthma by mid-adulthood, with an association between severity, sensitization, and risk of persistence [15, 16]. Considering child growth and development as well as environmental risk factors, asthma-related factors in preschool and school-aged children could be different from those in other age groups.

The purpose of this study was to explore the risk factors related to asthma prevalence among preschool and school-aged children according to sex using a representative national dataset from the general South Korean population.

## Methods

### Study population and database

We performed a cross-sectional study based on data obtained from the Korea National Health and Nutrition Examination Survey (KNHANES) conducted from 2009–2011 [17]. This nationwide survey has been assessing the health and nutrition status of Koreans since 1998 and each year includes approximately 10,000 individuals aged ≥1 year as a survey sample to collect information on socioeconomic status, health-related behaviors, quality of life, healthcare utilization, anthropometric measures, biochemical and clinical profiles for noncommunicable diseases, and dietary intakes with 3 component surveys: a health interview, health examination, and nutrition survey. The health interview and health examination are performed by trained medical staff and interviewers at a mobile examination center, and dieticians’ visits to the homes of the study participants are followed up [18]. The survey for children <12 years of age is answered by a parent or family member who lived with their child and was most aware of the child’s health status. The response rates for the Health Interview and Health Examination Survey were 79.2% in 2009, 77.5% in 2010, and 76.1% in 2011[17].

The target population of KNHANES comprises noninstitutionalized Korean citizens residing in Korea. The sampling plan follows a multi-stage clustered probability design. For example, in the 2011 survey, 92 primary sampling units (PSUs) were drawn from approximately 200,000 geographically defined PSUs for the whole country. A PSU consisted of an average of 60 households, and 20 final target households were sampled for each PSU using systematic sampling; in the selected households, all individuals aged ≥1 year were targeted [18]. The total numbers of households and individuals participating in the 2009–2011 surveys were 12,280 (4,600 in 2009, 3,840 in 2010, and 3,840 in 2011) and 28,009 (10,533 in 2009, 8,958 in 2010, and 8,518 in 2011), respectively. The 2009–2011 surveys included 3,542 children aged 4–12 years (1,111 in 2009, 1,214 in 2010, and 1,217 in 2011).

For our study, we evaluated the demographic information, health status, household environment, socioeconomic status, and parents’ health status of the 3,542 children aged 4–12 years. However, data obtained from 206 respondents were excluded because there was no information on their parents, and data obtained from 118 respondents were excluded owing to missing information for important questions (e.g., child’s asthma diagnosis). Therefore, the sample size was 3,218, which included 1,707 boys and 1,511 girls aged 4–12 years. Ethical approval from an Institutional Review Board was not required because the KNHANES is a publicly available dataset.

### Measurements

The questionnaire content related to our study was the same over the 3 years (2009–2011). The outcome variable was the physician-based asthma diagnostic status. Physician-diagnosed asthma was defined as a positive answer to the question, “Have you ever had asthma diagnosed by a physician in the past?”

To identify the demographic characteristics of the sample according to the asthma diagnostic status, data including age, sex, household income, number of family members, and residence were collected. The annual household income, which was based on reports from household heads during the Health Interview Survey, was chosen as a sociodemographic factor. The household income levels were divided into quartiles based on the national median household income. Considering the distribution of family size, the number of family members was classified into 3 categories: ≤3, 4, and ≥5 members. Place of residence was classified, according to the national administration system, as living in an urban area or not.

Obesity was determined according to body mass index (BMI), which was based on anthropometric measurements. To evaluate overweight and obesity prevalence according to BMI-for-age levels, overweight and obesity were defined as 85^th^ ≤ BMI < 95^th^ percentile and BMI ≥ 95^th^ percentile for age and sex, respectively, using the 2007 Korean Pediatric Growth Charts [19].

In accordance with a review of the literature on childhood asthma, data regarding asthma-related factors of comorbid atopic dermatitis, household smoking, parental asthma, maternal obesity, and maternal education level were also collected. Comorbid atopic dermatitis, household smoking, and maternal obesity were grouped into yes/no categories. Comorbid atopic dermatitis was defined as “yes” in children who had been diagnosed with atopic dermatitis by a physician and “no” in others. Household smoking was determined according to the parents’ responses, with “yes” indicating the presence of a daily household smoker and “no” indicating otherwise. Maternal obesity was defined as a BMI ≥25 kg/m^2^ for a child’s mother. Regarding parental asthma, as the study included only 2 cases in which both parents had been diagnosed with asthma, the subjects were divided into a group in which at least one parent had been diagnosed with asthma and another group in which neither parent had been diagnosed. Lastly, the maternal education level was divided as below or equal to high school completion versus equal to or above college completion.

### Data analysis

All statistics of this survey were calculated using sample weights assigned to sample participants. The sample weights were constructed for sample participants to represent the Korean population by accounting for the complex survey design, survey nonresponse, and post-stratification. The weights based on the inverse of selection probabilities and inverse of response rates were modified by adjusting them to the sex- and age-specific Korean populations (post-stratification) [18]. We calculated an integrated weight in proportion to the total number of PSUs of each year and built an integrated 3-year dataset (2009–2011) by applying this integrated weight. This integrated dataset was used in all analyses [17].

Based on a review of the literature, we first selected childhood asthma-related factors including age, sex, overweight/obesity prevalence, household income, place of residence, number of family members, family generation context, comorbid atopic dermatitis, maternal/paternal age, maternal/paternal education level, maternal/paternal occupation, maternal/paternal obesity, maternal/paternal asthma history, and household smoking from the 3-year dataset. To identify differences between the asthma and nonasthma groups, χ^2^ tests (PROC SURVEYFREQ in SAS) were used for the categorical variables chosen above, and a t-test (PROC SURVEYREG) was conducted for a continuous variable. As a result, the factors showing a statistically significant difference (p < 0.05) were chosen for multivariate logistic regression, provided that basic demographic factors, overweight/obesity prevalence, maternal obesity, maternal education, and exposure to household smoking were included, irrespective of their p-values.

The general characteristics of the study population are displayed as weighted percentages for categorical variables and weighted mean with standard error of the mean for a continuous variable. A sex-stratified multivariate logistic regression (PROC SURVEYLOGISTIC in SAS) was used to obtain adjusted prevalence odds ratios (ORs) and 95% confidence intervals (CIs) after accounting for primary sample units, stratification, and sample weights from the KNHANES. Statistical analyses were performed using SAS software, version 9.2 (SAS Institute, Cary, NC, USA), and a p-value < 0.05 was considered statistically significant.

## Results

### General characteristics

Table 1 presents the general characteristics of the study participants according to sex. Boys had a higher asthma prevalence than girls (boys, 7.4%; girls, 6.3%), and there was a higher proportion of girls with comorbid atopic dermatitis than there was of boys (boys, 14.7%; girls, 17.5%). However, these differences were not statistically significant. In addition, the proportion of children with at least one asthmatic parent was higher among boys than among girls (boys, 5.9%; girls, 3.3%, p = 0.0131), and the proportion of children living in an urban area was higher among girls than among boys (boys, 82.1%; girls, 85.3%, p = 0.0462).

**Table 1:** Sample Characteristics.

Variable	Boys (n = 1,707)	Girls (n = 1,511)	All (n = 3,218)
	% or M(SE)	% or M(SE)	% or M(SE)
Age (years)	8.33 (0.08)	8.25 (0.08)	8.29 (0.06)
Household income quartile			
Lowest	9.12	8.58	8.87
Middle-low	31.22	31.59	31.39
Middle-high	33.10	34.74	33.86
Highest	26.56	25.08	25.88
Number of family members			
≤ 3 members	12.56	12.61	12.58
4 members	53.58	52.41	53.04
≥ 5 members	33.86	34.98	34.38
Residence			
Urban area	82.05	85.28	83.54
Rural area	17.95	14.72	16.46
Household smoking			
Yes	24.01	22.10	23.13
No	75.99	77.90	76.87
Overweight/Obesity			
Yes	19.42	17.12	18.36
No	80.58	82.88	81.64
Diagnosis of asthma			
Yes	7.39	6.27	6.87
No	92.61	93.73	93.13
Diagnosis of atopic dermatitis			
Yes	14.65	17.51	15.97
No	85.35	82.49	84.03
Parental asthma			
Some parent asthma	5.86	3.29	4.68
Neither parent asthma	94.14	96.71	95.32
Maternal obesity			
Yes	23.51	22.03	22.82
No	76.50	77.97	77.18
Paternal obesity			
Yes	43.17	39.17	41.37
No	56.83	60.83	58.63
Maternal educational level			
≤ Middle school	6.23	4.68	5.52
High school	51.71	52.22	51.94
≥ College	42.06	43.10	42.54
Paternal educational level			
≤ Middle school	8.14	7.38	7.80
High school	40.26	40.28	40.27
≥ College	51.59	52.34	51.93

Results are presented as weighted percentages using the survey sample weights for categorical variables, and weighted mean (standard error of the mean) for the continuous variable age.

### Comparison of related factors between asthma and nonasthma groups by sex

Table 2 shows the differences in asthma-related factors between asthma and nonasthma groups, stratified by sex. The proportion of boys with comorbid atopic dermatitis was higher among those with asthma than those without asthma (27.3% vs. 13.6%, respectively; p = 0.0008), and a similar result was observed in girls (30.0% vs. 16.7%, respectively; p = 0.0078). There was a 12.4% higher proportion of boys with asthma had who had at least one asthmatic parent compared with boys without asthma (17.3% vs. 4.9%, respectively; p < 0.0001). Meanwhile, 11.0% of girls with asthma had at least one asthmatic parent, compared with 2.8% of those without asthma (p = 0.0013). There was a 9.5% lower proportion of girls with asthma living in a rural area compared with girls without asthma (5.9% vs. 15.3%, respectively; p = 0.0078).

**Table 2:** Comparison of related factors between asthma and nonasthma groups, stratified by sex.

Variable	Boys(n = 1,707)		p-value	Girls(n = 1,511)		p-value
	Diagnosis of asthma			Diagnosis of asthma		
	Yes	No		Yes	No	
	% or M(SE)	% or M(SE)		% or M(SE)	% or M(SE)	
Age (years)	8.12 (0.08)	8.34 (0.08)	0.4478	7.80 (0.32)	8.28 (0.08)	0.1475
Household income quartile						
Lowest	9.19	9.12	0.7345	9.66	8.51	0.1790
Middle-low	28.63	31.42		37.10	31.23	
Middle-high	30.61	33.30		21.33	35.63	
Highest	31.57	26.16		31.90	24.63	
Number of family members						
≤ 3 members	6.36	13.05	0.1635	17.27	12.30	0.5329
4 members	54.63	53.50		48.43	52.68	
≥ 5 members	39.00	33.45		34.31	35.02	
Residence						
Urban area	82.04	82.05	0.9994	94.15	84.69	**0.0078**
Rural area	17.96	17.95		5.85	15.31	
Household smoking						
Yes	22.43	24.14	0.7345	22.02	22.10	0.9885
No	77.57	75.86		77.98	77.90	
Overweight/Obesity						
Yes	19.53	19.42	0.9785	13.83	17.34	0.4066
No	80.47	80.58		86.17	82.66	
Diagnosis of atopic dermatitis						
Yes	27.32	13.64	**0.0008**	29.97	16.68	**0.0078**
No	72.68	86.36		70.03	83.32	
Parental asthma						
Some parent asthma	17.32	4.94	**<.0001**	11.02	2.78	**0.0013**
Neither parent asthma	82.68	95.06		88.98	97.22	
Maternal obesity						
Yes	30.10	22.96	0.1963	27.17	21.67	0.3600
No	69.90	77.04		72.83	78.33	
Maternal educational level						
≤ Middle school	2.18	6.57	0.2115	9.70	4.34	0.1431
High school	50.43	51.81		44.04	52.78	
≥ College	47.39	41.62		46.26	42.88	

Results are presented as weighted percentages using the survey sample weights for categorical variables, and weighted mean (standard error of the mean) for the continuous variable age. The p-values were obtained using χ^2^ tests for categorical variables and a t-test for the continuous variable age.

### Differences in asthma-related factors according to sex

The ORs and 95% CIs for asthma prevalence according to sex are shown in Table 3. Asthma prevalence was significantly associated with comorbid atopic dermatitis, parental history of asthma, and number of family members among boys. Meanwhile, age, residence, atopic dermatitis, and parental asthma were related to asthma prevalence among girls.

**Table 3:** Factors associated with asthma prevalence from sex-stratified multivariate logistic regression^a^.

Variable	Boys (n = 1,707)	Girls (n = 1,511)
	OR (95%CI)	OR (95%CI)
Age	0.97 (0.889–1.056)	**0.90 (0.809–0.996)** *
Household income quartile		
Lowest (reference)		
Middle-low	0.83 (0.336–2.062)	0.97 (0.371–2.507)
Middle-high	0.81 (0.318–2.083)	0.49 (0.177–1.354)
Highest	1.14 (0.432–2.995)	0.95 (0.370–2.412)
Number of family members		
≤ 3 members (reference)		
4 members	2.12 (0.977–4.592)	0.81 (0.386–1.707)
≥ 5 members	**2.45 (1.079–5.570)** *	0.83 (0.361–1.894)
Residence		
Urban area (reference)		
Rural area	0.98 (0.497–1.916)	**0.40 (0.174–0.919)** *
Household smoking		
No (reference)		
Yes	0.83 (0.449–1.531)	1.08 (0.571–2.042)
Overweight/Obesity		
No (reference)		
Yes	1.10 (0.633–1.922)	0.75 (0.365–1.532)
Atopic dermatitis		
No (reference)		
Yes	**2.20 (1.239–3.892)** **	**2.33 (1.330–4.064)** **
Parental asthma		
Neither parent asthma (reference)		
Some parent asthma	**3.90 (1.785–8.521)** ***	**3.65 (1.303–10.219)** *
Maternal obesity		
No (reference)		
Yes	1.43 (0.824–2.495)	1.47 (0.760–2.839)
Maternal educational level		
≤ High school (reference)		
≥ College	1.21 (0.746–1.970)	0.99 (0.554–1.758)

Results are presented as odds ratios (95% confidence intervals).

^a^Asterisks indicate significant results:

*p < 0.05;

**p < 0.01;

***p < 0.001.

As girls got older, the prevalence of asthma decreased (OR = 0.90, p = 0.0408). Girls residing in rural areas were 60% less likely to have asthma than those residing in urban areas (p = 0.0309). Boys and girls with comorbid atopic dermatitis were more likely to have asthma than those without atopic dermatitis (boys: OR = 2.20, p = 0.0071; girls: OR = 2.33, p = 0.0031). Boys and girls with at least one asthmatic parent were more likely to have asthma than those without asthmatic parents (boys: OR = 3.90, p = 0.0006; girls: OR = 3.65, p = 0.0138). The number of family members had significant effects only among boys. Boys with ≥5 family members were more likely to have asthma than those with ≤3 family members (OR = 2.45, p = 0.0323). However, when a Bonferroni correction is used to evaluate the multiple comparisons, p-values < 0.025 would be considered statistically significant. Therefore, caution is needed in interpretation of our findings on asthma-related factors such as age, residence, and number of family members.

## Discussion

In this study, we identified factors related to asthma prevalence among preschool and school-aged children according to sex. There are some key findings in this study. First, age was related to asthma prevalence in girls. The older girls had lower asthma prevalence. Second, obesity was not associated with the asthma prevalence in boys or girls. Third, the asthma prevalence in rural areas was significantly lower among girls, but not among boys. Fourth, the asthma prevalence was higher in boys with a larger family size. Lastly, comorbid atopic dermatitis and parental asthma were predictors of asthma for both sexes.

Although age was significantly associated with the asthma prevalence in girls only, there was a trend of lower asthma prevalence in older boys and girls in this study. A similar pattern was presented in previous studies. Broms and colleagues [20] reported that the prevalence of asthma was decreased by age among children 1–6 years of age. Another recent study also showed that the asthma prevalence in girls after late puberty was significantly lower than that in younger girls [21]. Generally, it is known that asthma is more common and more severe in prepubertal boys than girls of the same age, but the prevalence of asthma and its severity increases significantly in women after puberty [13, 14]. The mean age of this study sample was 8.29 years. Thus, our results support that the asthma prevalence might be decreased with advancing years in these prepubertal children.

We could not find an association between obesity and asthma prevalence in our study. Previous studies revealed that BMI was an important asthma-related factor among children, especially girls [13, 22]. Other studies explained a relationship between obesity and asthma in women starting in adolescence as possibly due to the effect of sex hormones [23, 24]. However, another study showed obesity was not associated with asthma prevalence in preschool children [25]. The findings seem to be dependent on the severity of obesity. Obesity is related to asthma prevalence in both adults and children, but overweight was not significantly related to asthma in previous studies targeting adults and children [25–27]. Therefore, further studies are required to provide a better understanding of the association between obesity and asthma in a large sample of children.

The place of residence was a significant variable among girls, but not among boys, in this study. Girls who resided in rural areas had lower asthma prevalence relative to those residing in urban areas. Consistent with our finding, a previous study reported that school-aged children residing in rural areas tended to have lower asthma prevalence than those in urban areas [10]. However, very few previous studies investigated the association between place of residence and asthma prevalence according to sex. When children begin to attend school, they suddenly experience exposure to the outdoor environment, and generally, boys are more physically active than girls [28, 29]. The association between physical activity and asthma has been studied; a higher asthma prevalence is associated with higher physical activity [30]. The sex-based difference in asthma prevalence according geographic region might be related to the difference in the amount of physical activity between boys and girls. However, as the difference in asthma prevalence according to sex is still unclear, and the difference between boys and girls in the place of residence appeared in our sample, further studies are required.

The association between family size and asthma prevalence seems unclear in this study. We found that the number of family members was not associated with the asthma prevalence among girls, but the boys having more family members showed more asthma prevalence. A previous study reported a greater prevalence of more severe asthma symptoms in larger families in children aged 6–7 years [31]. However, other study showed that a larger family size was related to lower asthma prevalence in children aged 7–9 years [32]. The family size was defined as the total number of family members in our study rather than the number of siblings. Because our data were derived from a nationwide dataset, there were limitations to our ability to obtain information such as birth order and family culture. This uncertainty might tend to attenuate the true relationship between family size and asthma prevalence in this sample.

Our study supports the relationship between asthma prevalence and atopic dermatitis and parental asthma in both boys and girls. As the proportion of children with at least one asthmatic parent in our sample was higher among boys, the relationship in boys seems to be more significant than in girls. These relationships are well known in subjects of all ages. Studies have reported that atopic dermatitis was associated with asthma prevalence among children [33, 34] and that parental asthma was a predictor of wheeze for both sexes [24].

The relationship between maternal education level and asthma prevalence seems unclear. In our study, maternal education level was not associated with asthma prevalence among boys and girls. The studies from western countries showed that children with a low socioeconomic status or parental education level had a higher risk of asthma [8, 9]. However, a Taiwanese study found that as the parental education levels increased, the incidence of asthma attacks among children younger than 12 years old increased [35]. In addition, a study in the United States found an association between a higher maternal education level and higher asthma prevalence only among 13- to 17-year-old adolescents [36]. However, other study showed no association between socioeconomic status and asthma incidence [37]. The parents’ ability to perceive their child’s health status may be directly connected to the child’s access to healthcare. In that respect, educating parents with school-aged children about asthma might have an important impact on asthma prevention and treatment. Further study is needed to explore the association between maternal education level and asthma prevalence while controlling for other environmental factors.

There are some strengths in our study. First, KNHANES is a continuous survey with nationally representative samples of Koreans that have been used for the development of Korean standards regarding health and nutrition. The data have also been used in international comparison studies. All children aged 4–12 years who participated in the 2009–2011 KNHANES were chosen as our study participants. In addition, data from the study subjects’ parents were secured from the same database and were subsequently matched and analyzed. Therefore, the study results could also be nationally representative. Second, because this study examined the association between risk factors and the asthma prevalence according to sex, effective interventional strategies could be devised based on the sex-based groups. To further investigate sex differences with respect to the association between genetic and environmental factors, a serial assessment from before birth to adulthood may be needed. In addition, a strength of this study is that the findings may suggest the importance of conducting a nationwide prospective birth cohort study.

There are several limitations in this study. First, the Health Interview Survey data from the National Health and Nutrition Examination Survey were acquired using self-reporting survey tools, and therefore, the data may be subjective and could differ from data obtained during a professional evaluation. Second, an objective analysis of the physiological asthma diagnosis and asthma severity data was not conducted. An additional limitation to this study is its cross-sectional design, and caution is needed in the interpretation of the associations. Finally, although data from the last 3 years were combined for the analysis, the sample size might be insufficient to assume the generalizability of the results.

## Conclusions

In this study targeting preschool and school-aged children, we found that factors related to asthma prevalence may differ depending on sex. The parental history of asthma and atopic dermatitis were significantly associated with asthma prevalence in both boys and girls. Age and residence were found to be associated with asthma prevalence among girls. The number of family members was a risk factor of asthma among boys. Based on understanding the characteristics of sex-based differences in asthma, individualized asthma management plans according to sex may be established clinically. In addition, appropriate educational programs regarding asthma prevention and management may be provided depending on sex in preschool and school-aged children. Furthermore, by detecting and treating childhood asthma, the persistence of asthma into adulthood could be minimized, thus ultimately leading to a reduced socioeconomic burden on patients as well as an increased quality of life.

## References

[1] World Health Organization. Asthma 2014. Available: http://www.who.int/respiratory/asthma/en/. Accessed 2014 Nov 14.

[2] Asher MI, Montefort S, Bjorksten B, Lai CK, Strachan DP, Weiland SK, et al Worldwide time trends in the prevalence of symptoms of asthma, allergic rhinoconjunctivitis, and eczema in childhood: ISAAC Phases One and Three repeat multicountry cross-sectional surveys. Lancet. 2006;368(9537):733–43. 16935684 10.1016/S0140-6736(06)69283-0

[3] Korea Centers for Disease Control and Prevention. Guidelines for prevention and control of asthma. 2013. Available: http://atopy.cdc.go.kr/atopy/index.do. Accessed 2014 Oct 31.

[4] Oh I-H, Yoon S-J, Kim E-J. The burden of disease in Korea. J Korean Med Assoc. 2011;54(6):646–52.

[5] Yoon SJ, Bae SC, Lee SI, Chang H, Jo HS, Sung JH, et al Measuring the burden of disease in Korea. J Korean Med Sci. 2007;22(3):518–23. 17596664 10.3346/jkms.2007.22.3.518 PMC2693648

[6] Korea Centers for Disease Control and Prevention. 2011 Korean Youth Risk Behavior Web-based Survey In: Prevention KCfDCa, editor.: Ministry of Health and Welfare of Korea; 2012.

[7] Chen E, Hanson MD, Paterson LQ, Griffin MJ, Walker HA, Miller GE. Socioeconomic status and inflammatory processes in childhood asthma: the role of psychological stress. J Allergy Clin Immunol. 2006;117(5):1014–20. 16675327 10.1016/j.jaci.2006.01.036

[8] Clement LT, Jones CA, Cole J. Health disparities in the United States: childhood asthma. Am J Med Sci. 2008;335(4):260–5. 18461727 10.1097/maj.0b013e318169031c

[9] Crespo NC, Ayala GX, Vercammen-Grandjean CD, Slymen DJ, Elder JP. Sociodemographic disparities of childhood asthma. J Child Health Care. 2011;15(4):358–69. 10.1177/1367493510397680 21996682

[10] Lawson JA, Janssen I, Bruner MW, Madani K, Pickett W. Urban-rural differences in asthma prevalence among young people in Canada: the roles of health behaviors and obesity. Ann Allergy Asthma Immunol. 2011;107(3):220–8. 10.1016/j.anai.2011.06.014 21875540

[11] Heinrich J. Influence of indoor factors in dwellings on the development of childhood asthma. Int J Hyg Environ Health. 2011;214(1):1–25. 10.1016/j.ijheh.2010.08.009 20851050

[12] Wandalsen GF, Borges LV, Barroso N, Rota R, Suano F, Mallol J, et al Gender differences in the relationship between body mass index (BMI) changes and the prevalence and severity of wheezing and asthma in the first year of life. Allergol Immunopathol (Madr). 2015. 10.1016/j.aller.2014.10.002 25796306

[13] Almqvist C, Worm M, Leynaert B. Impact of gender on asthma in childhood and adolescence: a GA2LEN review. Allergy. 2008;63(1):47–57. 17822448 10.1111/j.1398-9995.2007.01524.x

[14] Vink NM, Postma DS, Schouten JP, Rosmalen JG, Boezen HM. Gender differences in asthma development and remission during transition through puberty: the TRacking Adolescents' Individual Lives Survey (TRAILS) study. J Allergy Clin Immunol. 2010;126(3):498–504.e1–6. 10.1016/j.jaci.2010.06.018 20816186

[15] Pyun B. Relationship between atopic dermatitis, wheezing during infancy and asthma development. Journal of Korean Medical Association 2007;50(6):533–8.

[16] Bisgaard H, Bonnelykke K. Long-term studies of the natural history of asthma in childhood. J Allergy Clin Immunol. 2010;126(2):187–97; quiz 98–9. 10.1016/j.jaci.2010.07.011 20688204

[17] Korea Centers for Disease Control and Prevention. Korean National Health and Nutrition Examination Survey. 2013. Available: http://knhanes.cdc.go.kr/. Accessed 2013 Jun 1.

[18] Kweon S, Kim Y, Jang MJ, Kim Y, Kim K, Choi S, et al Data resource profile: the Korea National Health and Nutrition Examination Survey (KNHANES). Int J Epidemiol. 2014;43(1):69–77. 10.1093/ije/dyt228 24585853 PMC3937975

[19] Moon J, Lee S, Nam C, Choi J, Choe B, Seo J, et al 2007 Korean National Growth Charts: review of developmental process and an outlook. Korean J Pediatr. 2008;51(1):1–25.

[20] Broms K, Norback D, Eriksson M, Sundelin C, Svardsudd K. Effect of degree of urbanisation on age and sex-specific asthma prevalence in Swedish preschool children. BMC Public Health. 2009;9:303 10.1186/1471-2458-9-303 19695101 PMC2741449

[21] Protudjer JL, Lundholm C, Bergstrom A, Kull I, Almqvist C. Puberty and asthma in a cohort of Swedish children. Ann Allergy Asthma Immunol. 2014;112(1):78–9. 10.1016/j.anai.2013.10.015 24331402

[22] Scholtens S, Wijga AH, Seidell JC, Brunekreef B, de Jongste JC, Gehring U, et al Overweight and changes in weight status during childhood in relation to asthma symptoms at 8 years of age. J Allergy Clin Immunol. 2009;123(6):1312–8.e2 10.1016/j.jaci.2009.02.029 19409606

[23] Hancox RJ, Milne BJ, Poulton R, Taylor DR, Greene JM, McLachlan CR, et al Sex differences in the relation between body mass index and asthma and atopy in a birth cohort. Am J Respir Crit Care Med. 2005;171(5):440–5. 15557135 10.1164/rccm.200405-623OC

[24] Tse SM, Coull BA, Sordillo JE, Datta S, Gold DR. Gender- and age-specific risk factors for wheeze from birth through adolescence. Pediatr Pulmonol. 2014. 10.1002/ppul.23113 PMC4800823 25348842

[25] Vazquez-Nava F, Morales Romero J, Crodova Fernandez JA, Saldivar-Gonzalez AH, Vazquez-Rodriguez CF, Barrientos Gomez Mdel C, et al Association between obesity and asthma in preschool Mexican children. ScientificWorldJournal. 2010;10:1339–46. 10.1100/tsw.2010.134 20623094 PMC5763817

[26] Chen YC, Dong GH, Lin KC, Lee YL. Gender difference of childhood overweight and obesity in predicting the risk of incident asthma: a systematic review and meta-analysis. Obes Rev. 2013;14(3):222–31. 10.1111/j.1467-789X.2012.01055.x 23145849

[27] Wang L, Wang K, Gao X, Paul TK, Cai J, Wang Y. Sex difference in the association between obesity and asthma in U.S. adults: Findings from a national study. Respir Med. 2015. 10.1016/j.rmed.2015.06.001 26118569

[28] Aarts MJ, de Vries SI, van Oers HA, Schuit AJ. Outdoor play among children in relation to neighborhood characteristics: a cross-sectional neighborhood observation study. Int J Behav Nutr Phys Act. 2012;9:98 10.1186/1479-5868-9-98 22901102 PMC3478217

[29] Gonzalez-Barcala FJ, Pertega S, Sampedro M, Lastres JS, Gonzalez MA, Bamonde L, et al Impact of parental smoking on childhood asthma. J Pediatr (Rio J). 2013;89(3):294–9. 23684453 10.1016/j.jped.2012.11.001

[30] Ownby DR, Peterson EL, Nelson D, Joseph CC, Williams LK, Johnson CC. The relationship of physical activity and percentage of body fat to the risk of asthma in 8- to 10-year-old children. J Asthma. 2007;44(10):885–9. 18097868 10.1080/02770900701752698

[31] Strachan DP, Ait-Khaled N, Foliaki S, Mallol J, Odhiambo J, Pearce N, et al Siblings, asthma, rhinoconjunctivitis and eczema: a worldwide perspective from the International Study of Asthma and Allergies in Childhood. Clin Exp Allergy. 2015;45(1):126–36. 10.1111/cea.12349 24912652 PMC4298795

[32] Genuneit J, Strachan DP, Buchele G, Weber J, Loss G, Sozanska B, et al The combined effects of family size and farm exposure on childhood hay fever and atopy. Pediatr Allergy Immunol. 2013;24(3):293–8. 10.1111/pai.12053 23551831

[33] Cho YM, Ryu SH, Choi MS, Tinyami ET, Seo S, Choung JT, et al Asthma and allergic diseases in preschool children in Korea: findings from the pilot study of the Korean Surveillance System for Childhood Asthma. J Asthma. 2014;51(4):373–9. 10.3109/02770903.2013.876648 24393081

[34] Zheng T, Yu J, Oh MH, Zhu Z. The atopic march: progression from atopic dermatitis to allergic rhinitis and asthma. Allergy Asthma Immunol Res. 2011;3(2):67–73. 10.4168/aair.2011.3.2.67 21461244 PMC3062798

[35] Lee YL, Lin YC, Hwang BF, Guo YL. Changing prevalence of asthma in Taiwanese adolescents: two surveys 6 years apart. Pediatr Allergy Immunol. 2005;16(2):157–64. 15787874 10.1111/j.1399-3038.2005.00211.x

[36] Ahmad N, Biswas S, Bae S, Meador KE, Huang R, Singh KP. Association between obesity and asthma in US children and adolescents. J Asthma. 2009;46(7):642–6. 10.1080/02770900802503123 19728197

[37] Rodriguez-Martinez CE, Sossa-Briceno MP, Castro-Rodriguez JA. Predictors of hospitalization for asthma in children: results of a 1-year prospective study. Pediatr Pulmonol. 2014;49(11):1058–64. 10.1002/ppul.22936 24376022

